# Development of a Mouse Model of Prostate Cancer Using the Sleeping Beauty Transposon and Electroporation

**DOI:** 10.3390/molecules23061360

**Published:** 2018-06-05

**Authors:** Hyun-Ji Choi, Han-Byul Lee, Sunyoung Jung, Hyun-Kyu Park, Woori Jo, Sung-Min Cho, Woo-Jin Kim, Woo-Chan Son

**Affiliations:** 1 Asan Institute for Life Sciences, Asan Medical Center, Songpa-gu, 05505 Seoul, Korea; snapple08@naver.com (H.-J.C.); onestar0620@naver.com (H.-B.L.); flandus@naver.com (S.J.); nevok@hanmail.net (H.-K.P.); c2dar@hanmail.net (W.J.); somnium14@naver.com (S.-M.C.); 2 Department of Pathology, University of Ulsan College of Medicine, Songpa-gu, 05505 Seoul, Korea; woojinindi@naver.com

**Keywords:** electroporation, insertional mutagenesis, animal models, prostatic neoplasms, Sleeping Beauty transposase

## Abstract

The Sleeping Beauty (SB) transposon system is non-viral and uses insertional mutagenesis, resulting in the permanent expression of transferred genes. Although the SB transposon is a useful method for establishing a mouse tumor model, there has been difficulty in using this method to generate tumors in the prostate. In the present study, electroporation was used to enhance the transfection efficiency of the SB transposon. To generate tumors, three constructs (a *c-Myc* expression cassette, a *HRAS* (HRas proto-oncogene, GTPase) expression cassette and a shRNA against *p53*) contained within the SB transposon plasmids were directly injected into the prostate. Electroporation was conducted on the injection site after the injection of the DNA plasmid. Following the tumorigenesis, the tumors were monitored by animal PET imaging and identified by gross observation. After this, the tumors were characterized by using histological and immunohistochemical techniques. The expression of the targeted genes was analyzed by Real-Time qRT-PCR. All mice subjected to the injection were found to have prostate tumors, which was supported by PSA immunohistochemistry. To our knowledge, this is the first demonstration of tumor induction in the mouse prostate using the electroporation-enhanced SB transposon system in combination with *c-Myc*, *HRAS* and *p53*. This model serves as a valuable resource for the future development of SB-induced mouse models of cancer.

## 1. Introduction

Prostate cancer is the most common cancer among men worldwide. Nevertheless, there are currently no mouse models that fully recapitulate the features of human prostate cancer, as spontaneously occurring prostate cancer is uncommon in mice [1,2]. In a 2-year toxicology and carcinogenicity study conducted by the National Toxicology Program, 612 control B6C3F1 mice failed to develop neoplastic lesions [3]. Therefore, there is a great need for a mouse model of prostate cancer. In this study, we used the Sleeping Beauty (SB) transposon system combined with electroporation to establish a mouse model of prostate cancer.

The SB transposon, which was genetically engineered for the purpose of insertional mutagenesis [4], is a member of the Tc1/mariner family [5]. Transposons are DNA pieces that are flanked by terminal inverted repeats (TIRs), which have the ability to change their positions within the genome by a “cut-and-paste” mechanism called transposition [6]. The SB transposon system consists of two main components: (i) a transposon containing a gene expression cassette; and (ii) the transposase enzyme, which catalyzes the mobilization and reintegration of the transposon into the genomic DNA [7,8,9]. The integration of the SB transposon elements into the host genome results in the continuous expression of the transgene, which makes the SB transposon system suitable for the development of molecularly defined tumorigenesis models [10,11].

Although various types of SB transposon-induced tumors have been reported, several types of tumors, such as tumors of the lung, mammary gland and prostate, have not yet been generated with the SB transposon system in the mouse model [5,12]. Low insertional mutagenesis efficiency would be one of the factors that are preventing a successful model of these tumors from being created.

The delivery of transposon vectors into target cells can be conducted via viral methods and non-viral methods, which include electroporation, polycations, nanoparticles and hydrodynamic injection [13]. Viral methods can cause inflammatory immune responses [13], while the method using the hydrodynamic injection is limited by the need for a large volume of the vector and by poor expression efficiency in large animals [14]. Furthermore, the methods using polycations and nanoparticles result in lower transfection rates compared to the method using hydrodynamic injection [15]. However, electroporation is safe to use in mice due to the absence of immunogenesis, in addition to being less technically challenging and more efficient than other methods [16,17]. Thus, we decided to develop an electroporation protocol with enhanced transfection efficiency.

To induce the tumorigenesis of the prostate, three constructs (a *HRAS* (HRas proto-oncogene, GTPase) expression cassette, a *c-Myc* expression cassette and a short hairpin RNA sequence targeting *p53*) were sub-cloned into the SB transposon vectors. A mixture of the SB transposon vectors and an SB transposase vector were injected into the prostate of C57BL/6 mice, followed by electroporation. This method provides a more rapid approach compared to the conventional genetic engineering techniques.

## 2. Results

### 2.1. Tumor Observation

**Figure 1:**
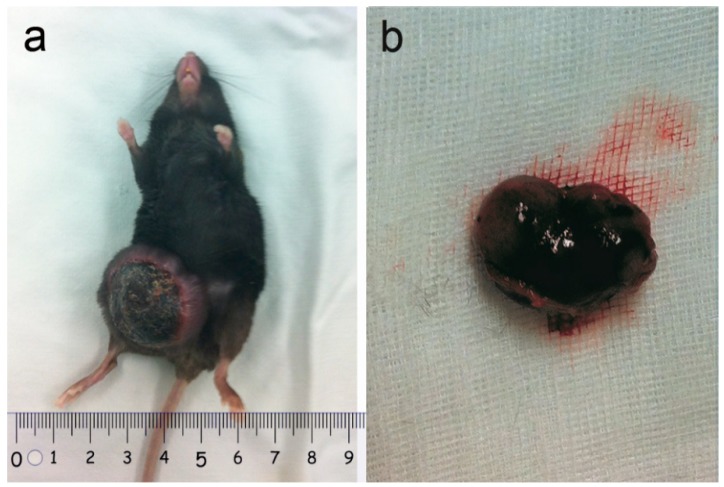
Tumor observation: (**a**) All recipients developed nodular neoplasms; (**b**) Each tumor showed single or coupled nodular features with a large volume.

All five recipients of the plasmid mixture (containing the three transposons and SB transposase) developed single or coupled nodular neoplasms approximately 3 weeks after injection (Figure 1).

### 2.2. Microscopic Findings

**Figure 2:**
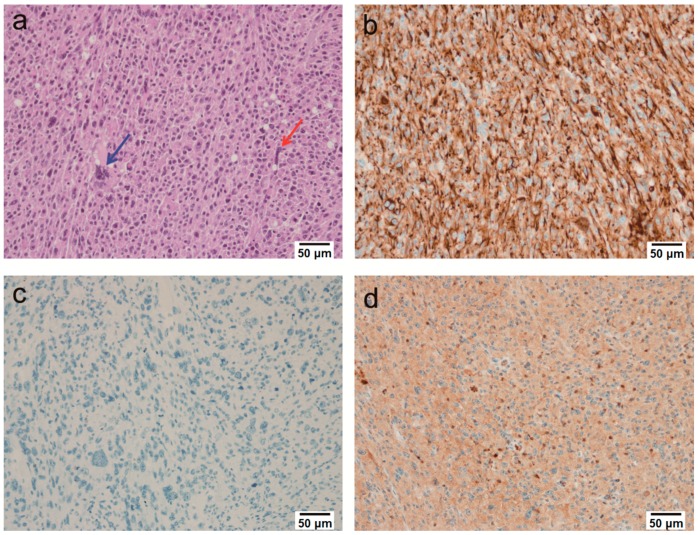
Microscopic analysis of prostate tumor tissue: (**a**) Tumors were highly undifferentiated and pleomorphic. Some of the tumor cells appeared to be epithelial or mesenchymal in origin. The poorly differentiated cells had a slightly basophilic cytoplasm and a round or oval shape. Multinucleated giant cells were occasionally visible (blue arrow). Bizarre cells with a large and pale cytoplasm and with prominent nuclei were also observed (red arrow) (H & E, 200×); (**b**) Positive immunoreactivity for pan-cytokeratin was seen (200×); (**c**) Immunostaining without the PSA primary antibody was negative 200×); (**d**) Immunostaining for PSA was positive (200×).

3 weeks after injection, the animals were euthanized, and then tumor nodules were extracted. The nodules were ovoid and well-demarcated at the sites of injection. Metastatic features were present at the injection sites, but there was no evidence of metastasis elsewhere. The tumors were undifferentiated, with high cellularity and both epithelial and mesenchymal components (Figure 2a). Abundant mitotic and apoptotic cells were observed. The tumor cells were pleomorphic, appearing to be round to oval in shape with pale basophilic cytoplasm and hyperchromatic nuclei with prominent nucleoli. Multinucleated giant cells and necrosis were also occasionally observed (Figure 2a). All tumors had the same morphological features.

### 2.3. Immunohistochemical Findings

Pan-cytokeratin was expressed in the tumors (Figure 2b), which suggested that the tumors were epithelial in origin. As other immunohistochemical markers were negative, including CD45, CD163, CD68, Desmin, Myogenin, Melanoma, S100, α-SMA, Cytokeratin 7, Cytokeratin 20, MDM2 and CDK4 (Table 1), the tumors were diagnosed as sarcomatoid carcinomas. In addition, the tumor tissues had positive results for the primary antibody of Prostate Specific Antigen (PSA) (Figure 2d), which suggested that these tumors originated from the prostate. Apart from the PSA primary antibody, the tumor had completely negative results with all other antibodies (Figure 2c).

### 2.4. Animal PET Imaging

**Figure 3:**
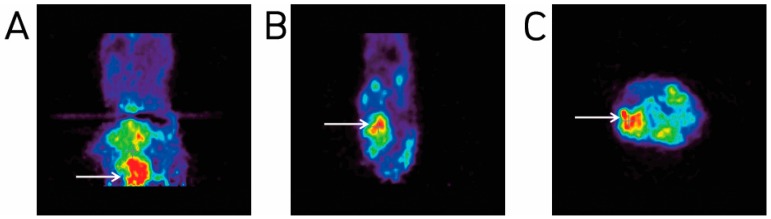
PET imaging of induced tumors: (**A**) Coronal; (**B**) sagittal and (**C**) transverse PET images of the injection sites show evidence of tumors. Tumors are shown in red (arrows).

The transverse and longitudinal PET imaging showed the presence of neoplasms (red/pink in color) at the injection sites (Figure 3).

### 2.5. Confirmation of Gene Expression Using Real-Time qRT-PCR

**Figure 4:**
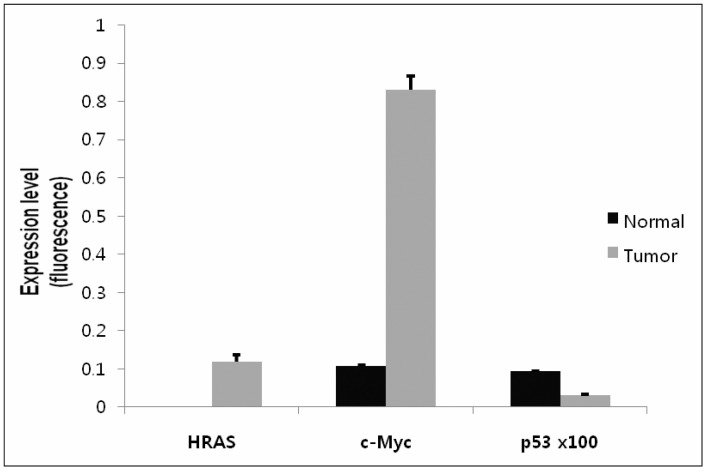
Analysis of gene expression by Real-Time qRT-PCR. The expression of the three targeted genes, *c-Myc*, *HRAS* and *p53*, was analyzed by Real-Time qRT-PCR. The graph shows the relative expression of the targeted genes in tumor and normal prostate tissues, which was normalized to the GAPDH internal control. Data represent mean ± standard deviation (SD) obtained from three independent experiments. 100 times the value of *p53* is displayed for representation in the same graph with *c-Myc* and *HRAS*.

The Real-Time qRT-PCR showed the successful expression of the transferred genes, *c-Myc* and *HRAS*. The expression of *HRAS* was increased in the tumor tissue compared to the normal prostate tissue, in which the *HRAS* expression was too low to be measured. The expression of *c-Myc* was more than 8-fold higher in the tumor tissue than in the normal prostate tissue. Finally, *p53* in the tumor tissue was decreased compared to the normal prostate tissue, which demonstrated its successful knock-out with the *p53* shRNA (Figure 4).

## 3. Discussion

As there is no current mouse model that fully recapitulates all the features of prostate cancer [2], we aimed to establish a transgenic mouse model of prostate cancer using the Sleeping Beauty transposon system enhanced by electroporation in this work.

Although retroviral insertional mutagenesis has been a powerful tool in the study of cancer in mice, their narrow cell type specificity is partially what prompted the development of the SB system for this application [8]. When the mobile elements of the SB transposons that encode the oncogenes or silence tumor suppressor genes are expressed simultaneously within a cell, tumorigenesis can be induced. Many tumors, including liver [10,12], intestine [18], pancreas [19] and skin [20,21] tumors, have been generated using the SB transposon system in mice or rats. Although the SB system has been used to induce many types of tumors in mice, investigators have had difficulty generating prostate cancers with the SB system [5]. Although one study used the SB transposon system to identify PDE4D as a candidate prostate cancer gene in mice based on the transposon insertion site [22], only focal epithelial proliferation and hyperplasia in the prostate were observed. We hypothesized that this was due to the low insertional mutagenesis efficiency in the prostate with the procedures which use embryos. Therefore, methods that are able to more efficiently deliver the SB transposon, such as electroporation, are required.

In electroporation, high-intensity electric pulses are delivered to the tissue, increasing the uptake of DNA into cells [23]. Under specific pulse conditions, the electroporation allows DNA to enter cells through the cell membrane by increasing the cell membrane permeability [24]. A study of the electroporation-mediated gene transfer to the swine heart showed that the gene expression was higher at sites that were electroporated following the injection of DNA compared to the sites that were injected without electroporation, which demonstrated the robustness of this approach [24]. In vivo electroporation is also a safe non-viral method for gene delivery, which results in a high gene delivery rate without inducing adverse immune responses [25]. As both the increased expression of oncogenes and decreased expression of tumor suppressor genes are required to induce tumor formation using the SB transposon system, the mice were given two transposon plasmids expressing oncogenes (*c-Myc* and *HRAS*) and one transposon plasmid containing a short hairpin RNA against the tumor suppressor gene *p53* [5]. In the present study, a mixture of these three transposon plasmids was directly injected into the prostate, along with a transposase-containing plasmid, before the injection site was electroporated.

Our results show that prostate tumors were generated at the sites that were electroporated and injected with SB transposons/SB transposase. The animal PET images and gross and microscopic pathologic findings support our findings of tumorigenesis. Commonly, the transgenic mouse models, which use embryos, and xenografts are used to generate in vivo cancer models. In the TRAMP (transgenic adenocarcinoma mouse prostate) model of prostate cancer, which uses the probasin promoter, focal adenocarcinomas do not develop until 10–20 weeks [26,27]. It is also time-consuming and technically challenging to generate the xenograft models of prostate cancer [28]. Furthermore, the tumors generated in this manner differ from human tumors with respect to the presence of stromal cells, the vascular and lymphatic structure and the presence of infiltrating immune cells [29]. However, in the present study, which used the SB transposon system in conjunction with electroporation transfection technology, tumors arose quickly from their own tissue at the sites of interest. In addition, diverse tumors could be induced by multiple gene combinations.

The tumors showed a high degree of cellularity, apoptosis and mitosis. The tumor cells were pleomorphic, with epithelial and mesenchymal features. They were round to oval in shape, with pale basophilic cytoplasm and hyperchromatic nuclei with prominent nucleoli. Multinucleated giant cells and necrotic areas were observed occasionally. All tumors had similar undifferentiated morphological features (Figure 2a). To clarify the origin of tumors, epithelial, muscular, hematopoietic cell, melanoma and adipose tissue markers were tested. In addition, to confirm whether the tumors were from prostate glands, a prostate specific marker PSA was examined. The tumors were negative for all immunohistochemical markers except pan-cytokeratin (Figure 2b) and PSA (Figure 2d), which suggested that they were epithelial tumors that originated from prostate glands. Consequently, these tumors were diagnosed as the sarcomatoid carcinomas of prostate. These results provide a novel method for the development of in vivo tumor models. Further studies of the genetic mechanism resulting specifically in the development of sarcomatoid carcinomas are needed.

There were no metastatic foci in the non-injected sites despite their malignant characteristics. In other studies, several transgenic mouse tumor models using the SB transposon system developed metastasis [18,19]. Recently, it was hypothesized that clonal selection could play a role in selecting the mutations associated with tumor metastasis [5]. In the SB transposon pancreas tumor model, the mutations in adherens junction and tight junction proteins, which are observed in SB-induced pancreatic cancer, are related to pancreatic tumor invasion and metastasis [19]. This finding suggests that in our model of SB-induced prostate cancer, there were no mutations associated with metastasis. Further studies are needed to clarify the relationship between the sites of insertion of the SB transposon and the occurrence of tumor metastasis.

The present study shows that the SB transposon system enhanced by electroporation induces tumors quickly and easily. A strong advantage of this method is its ability to induce tumorigenesis using the manufactured SB transposons containing known oncogenes (e.g., *c-Myc* and *HRAS*, which was used in this present study). As the tumors can be generated using specific genes of interest, this model can be extended to the study of anti-cancer therapeutics designed to target particular gene products.

To our knowledge, this is the first study that used the SB transposon system with electroporation to induce prostate tumors in combination with *c-Myc*, *HRAS* and *p53* in mice. As an adequate mouse model of prostate cancer has not been established, the SB transposon/electroporation system may provide the basis for new strategies in developing prostate cancer models. In addition, this SB-induced mouse cancer model could provide a platform for the identification of novel cancer genes and drug targets as well as for the preclinical testing of novel therapeutics [5,30]. Transient expression of the mutagenic genes might be constantly creating mutagenesis. Therefore, further study using targeted drug treatments on this model would give clues as to whether the insertional mutagenesis is a good model for cancer therapy or not.

## 4. Materials and Methods

### 4.1. Ethics Statement

All animal works were conducted according to the relevant national and international guidelines. All procedures and protocols were approved by the Institutional Animal Care and Use Committee (IACUC) of the Asan Institute for Life Sciences (IACUC No. 2012-13-139). Housing and experimental procedures were in accordance with the guidelines of the Institutional Animal Care and Use Committee of the Asan Institute for Life Sciences and relevant national and international guidelines.

### 4.2. Animals

Ten male, 5-week-old C57BL/6 mice were purchased from Orient Bio (Yongin, Korea). The mice used for the confirmation of tumorigenesis were later euthanized to investigate the tumors. The mice were housed at the laboratory animal facility at the Asan Institute for Life Sciences under specific pathogen-free conditions according to ICLAS (International Council for Laboratory Animal Science). Quarterly, the 4-week-old sentinel mice were exposed to dirty bedding to monitor the presence of microorganisms. The mice were free of viral pathogens (*Sendai virus*, *Mouse hepatitis virus, Ectromelia virus, Lymphocytic choriomeningitis virus*), bacterial pathogens (*Mycoplasma pulmonism*, *Clostridium piliforme*, *Bordetella bronchiseptica*, *Salmonella* spp., *Streptococcus pneumoniae*, *Pasteurella pneumotropica*, *Staphylococcus aureus*, *Citrobacter rodentium*) and parasitic pathogens (*Eimeria* spp., *Syphacia* spp., and *Ectoparacites*). Cages and bedding were sterilized and changed weekly. The room was maintained on a 12:12h light:dark cycle. The room temperature and humidity were maintained at 22 ± 2 °C and 55 ± 5%, respectively.

### 4.3. Plasmid Construction

*HRAS* or the *c-Myc* encoding cDNA was inserted into the pCXEGFP plasmid (kindly provided by Dr. Masaru Okabe of Osaka University, Osaka, Japan). After this, the transcriptional cassettes were cloned into a PT2/BH transposon vector (a generous gift of Drs. David Largaespada and Perry Hackett of the University of Minnesota, MN, USA). The PT2/shp53/GFP4 transposon plasmid, which encodes a short hairpin RNA against the tumor suppressor *p53*, was a kind gift from Dr. John Ohlfest at the University of Minnesota. The DNA for injection was purified using the EndoFree Plasmid Maxi kit (Qiagen, Germantown, MD, USA) in accordance with the manufacturer’s instructions.

### 4.4. DNA Plasmid Injection

Each mouse was injected with a mixture of the three transposons and an SB transposase plasmid (pPGK/SB13; kindly provided by Drs. David Largaespada and Perry Hackett of the University of Minnesota, MN, USA). SB transposase enzyme have been generated by site directed polymerase chain reaction, SB13 which pertains to K33A and T83A mutations in the original *SB10* gene (first described by Yant et al.), was expressed under the control of the PGK promoter in pPGK-SB13 [30,31,32]. The molar ratio of the transposase-encoding plasmid to transposon-containing plasmid was 1:2. The three transposons (total of 50 µg) were mixed in an equimolar ratio with 50 µL of phosphate-buffered saline that contained the transposase-encoding plasmid. An insulin syringe (31 G) was used to inject the mixture directly into the prostate. To calculate the molar ratio, the lengths of the transposon and transposase genes were rounded to the nearest kb (7000 kb and 5000 kb, respectively).

### 4.5. In Vivo Electroporation

Electroporation was conducted at the sites of injection of the plasmids. The area overlying the injection site was shaved to expose the skin. A 2 cm incision was made ventrally to the prostate and the injection site was electroporated with Cellectra (VGX International, Seoul, Korea and Inovio Pharmaceuticals, Blue Bell, PA, USA) containing three needle probes at 0.2 A for 4 s (three pulses at a pulse duration = 52 ms/pulse with 1-s intervals between pulses). This was performed in accordance with the manufacturer’s guidelines. The incision was closed with a subcuticular suture.

### 4.6. Radiopharmaceutical Preparation and Animal PET Imaging

The decay-corrected radiochemical yields ranged from 60% to 70%. Following the high-performance liquid chromatography (HPLC) purification, the radiochemical purity was 98 ± 1.2% (mean ± standard deviation (SD)). The specific activity of the [^18^F]Flu-deoxyglucose (FDG) obtained was greater than 100 TBq/mmol. The PET scans were performed using a microPET Focus 120 system (Concorde Microsystems, Knoxville, TN, USA) with resolutions of 1.18 mm (radial), 1.13 mm (tangential) and 1.44 mm (axial) from the center of the field of view. A total of 7.4 MBq (0.2 mCi) and 37 MBq (1 mCi) of the [^18^F]FDG was injected into the tail vein of each mouse, before 10-min static PET scans were obtained. Each mouse was anesthetized with isoflurane during the uptake and scanning periods. Heating tools were used to maintain the body temperature of the mice at 37 °C. The PET images were reconstructed by ordered subset expectation maximization in 2 dimensions (OSEM2D) using a cut-off frequency of 0.5 cycles per pixel. No attenuation correction was applied.

### 4.7. Histology and Immunohistochemistry

Following the macroscopic examination, the resected tumor tissues were fixed in 10% neutral buffered formalin. After this, the tissues were embedded in paraffin blocks and cut into 3-µm thick sections using a microtome. Hematoxylin and eosin (H & E) staining was performed according to standard protocols. Immunohistochemistry was carried out using an automated slide preparation system Benchmark XT (Ventana Medical systems Inc., Tucson, AZ, USA). Deparaffinization, epitope retrieval and immunostaining were performed according to the manufacturer’s instructions by using the cell conditioning solutions (CC1) and the BMK ultraVIEW diaminobenzidine (DAB) detection system (Ventana Medical Systems). The tumor sections were stained with pan-cytokeratin (1:3000), Cytokeratin 7 (1:1000), Cytokeratin 20 (1:1000), Myogenin (1:1000), Desmin (1:4000), α-smooth muscle actin (1:1000), CK45 (1:2000), CD163 (1:2000), CD68 (1:2000), Melanoma (1:100), S100 (1:400), CDK4 (1:1000), MDM2 (1:1000) and PSA (1:100). The positive signals were amplified using ultra-VIEW copper, before the sections were counterstained with hematoxylin and bluing reagent.

### 4.8. Detection of Gene Expression Using Quantitative Real Time-qRT-PCR

The expression of the targeted genes (*c-Myc*, *HRAS* and *p53*) was analyzed by Real-Time qRT-PCR PCR. The samples were obtained from the prostate tumor tissue and normal prostate tissue. The Arcturus Paradise Whole Transcript RT Reagent System (Applied Biosystems/Life Technologies, Grand Island, NY, USA) was used for the RNA isolation and reverse transcription of the samples. All PCR reactions were performed in a Light cycler 2.0 (Roche Applied Science, Indianapolis, IN, USA) according to the standard procedures [10]. The primers (all purchased from Roche Diagnostics, Mannheim, Germany) used for quantification of the transcript levels are as follows: *c-Myc* forward, TCC TGT ACC TCG TCC GAT TC and *c-Myc* reverse, GGA GGA CAG CAG CGA GTC; *HRAS* forward, GGA CGA ATA CGA CCC CAC TAT and *HRAS* reverse, TGT CCA ACA GGC ACG TCT C; *p53* forward, TAA AGG ATG CCC GTG CTG and *p53* reverse, TCT TGG TCT TCG GGT AGC TG. The probes (#12, #38 and #94) were purchased individually from the Universal Probe Library (Roche Applied Science). The mouse GAPDH (forward primer, GAG CCA AAC GGG TCA TCA and reverse primer, CAT ATT TCT CGT GGT TCA CAC C) was used with a Taqman probe (TIB MOLBIOL, Berlin, Germany) as a reference to normalize gene expression. For reproducibility, the experiments were performed in triplicate.

## Figures and Tables

**Table 1:** Antibodies used for immunohistochemistry.

Tissue Marker	IHC Antibody	Type	Dilution	Reactivity
Epithelial tissue marker	Pan-cytokeratin	M	1:3000	+
	Cytokeratin 7	P	1:1000	−
	Cytokeratin 20	M	1:1000	−
Muscular tissue marker	Myogenin	M	1:1000	−
	Desmin	M	1:4000	−
	α-smooth muscle actin	M	1:1000	−
Hematopoietic cell marker	CD45	M	1:2000	−
	CD163	M	1:2000	−
	CD68	M	1:2000	−
Melanoma marker	Melanoma	M	1:100	−
	S100	M	1:400	−
Adipose tissue marker	CDK4	M	1:1000	−
	MDM2	M	1:1000	−
Prostate marker	PSA	P	1:100	+

M, monoclonal; P, polyclonal; + is positive; − is negative.
